# Codon Usage Heterogeneity in the Multipartite Prokaryote Genome: Selection-Based Coding Bias Associated with Gene Location, Expression Level, and Ancestry

**DOI:** 10.1128/mBio.00505-19

**Published:** 2019-05-28

**Authors:** J. L. López, M. J. Lozano, A. Lagares, M. L. Fabre, W. O. Draghi, M. F. Del Papa, M. Pistorio, A. Becker, D. Wibberg, A. Schlüter, A. Pühler, J. Blom, A. Goesmann, A. Lagares

**Affiliations:** a IBBM—Instituto de Biotecnología y Biología Molecular, CONICET, CCT-La Plata, Departamento de Ciencias Biológicas, Facultad de Ciencias Exactas, Universidad Nacional de La Plata, La Plata, Argentina; b Laboratorio de Bioquímica, Microbiología e Interacciones Biológicas en el Suelo, Universidad Nacional de Quilmes-CONICET, Bernal, Argentina; c LOEWE Center for Synthetic Microbiology (SYNMIKRO), Philipps-Universität Marburg, Marburg, Germany; d CeBiTec—Centrum für Biotechnologie, Universität Bielefeld, Bielefeld, Germany; e Institute for Bioinformatics and Systems Biology, Justus-Liebig-Universität Giessen, Giessen, Germany; University of Maryland, School of Medicine

**Keywords:** codon usage, genome evolution, host-microbe interaction, mobile genetic elements, plasmidome

## Abstract

Prokaryotes represent an ancestral lineage in the tree of life and constitute optimal resources for investigating the evolution of genomes in unicellular organisms. Many bacterial species possess multipartite genomes offering opportunities to study functional variations among replicons, how and where new genes integrate into a genome, and how genetic information within a lineage becomes encoded and evolves. To analyze these issues, we focused on the model soil bacterium Sinorhizobium meliloti, which harbors a chromosome, a chromid (pSymB), a megaplasmid (pSymA), and, in many strains, one or more accessory plasmids. The analysis of several genomes, together with 1.4 Mb of accessory plasmid DNA that we purified and sequenced, revealed clearly different functional profiles associated with each genomic entity. pSymA, in particular, exhibited remarkable interstrain variation and a high density of singletons (unique, exclusive genes) featuring functionalities and modal codon usages that were very similar to those of the plasmidome. All this evidence reinforces the idea of a close relationship between pSymA and the plasmidome. Correspondence analyses revealed that adaptation of codon usages to the translational machinery increased from plasmidome to pSymA to pSymB to chromosome, corresponding as such to the ancestry of each replicon in the lineage. We demonstrated that chromosomal core genes gradually adapted to the translational machinery, reminiscent of observations in several bacterial taxa for genes with high expression levels. Such findings indicate a previously undiscovered codon usage adaptation associated with the chromosomal core information that likely operates to improve bacterial fitness. We present a comprehensive model illustrating the central findings described here, discussed in the context of the changes occurring during the evolution of a multipartite prokaryote genome.

## Abstract (precis)

Bacterial genomes usually include many thousands of genes which are expressed with diverse spatial-temporal patterns and intensities. A well-known evidence is that highly expressed genes, such as the ribosomal and other translation-related proteins (RTRPs), have accommodated their codon usage to optimize translation efficiency and accuracy. Using a bioinformatic approach, we identify core-genes sets with different ancestries, and demonstrate that selection processes that optimize codon usage are not restricted to RTRPs but extended at a genome-wide scale. Such findings highlight, for the first time, a previously undiscovered adaptation strategy associated with the chromosomal-core information. Contrasted with the translationally more adapted genes, singletons (i.e., exclusive genes, including those of the plasmidome) appear as the gene pool with the less-ameliorated codon usage in the lineage. A comprehensive summary describing the inter- and intra-replicon heterogeneity of codon usages in a complex prokaryote genome is presented.

## INTRODUCTION

Bacterial genomes are composed of a set of ancestral core genes that are conserved within the species and that mainly encode essential (i.e., so-called “housekeeping”) products along with a lower number of strain-specific and/or lineage-specific genes (i.e., unique, exclusive genes, referred to as “singletons”) that are thought to correspond to the more recently acquired and more highly strain-specific genetic information encoding accessory functions (1). Although most core genetic information is vertically inherited, in some instances, plasmids, phages, and other mobile genetic elements can be acquired via their horizontal transfer (2). Such genetic inputs constitute part of a mobile intraspecific and interspecific gene pool and represent an active source of putative novel functions that in prokaryotes favor innovative adaptive strategies in the face of changing environments (3).

Rhizobia are Gram-negative bacteria that populate soils worldwide, associate symbiotically with leguminous plants, and in many bacterial species—such as Sinorhizobium meliloti—bear a significant proportion of their genomes as large-sized plasmids (megaplasmids) and also as (functionally) cryptic plasmids that are intermediate to small in molecular size (4–6). All these complex features characterizing the multipartite rhizobial genomes make those rhizobia excellent models for investigating the dynamics and adaptation of genetic information at the evolutionary, functional, and nucleotide sequence levels, both within and among the different types of replicons. Understanding the evolution and plasticity of bacterial genomes among their different genetic components requires an elucidation of the vertical changes that have operated over generations as well as the processes associated with the horizontal acquisition of new genes. To that end, codon usage analysis has frequently been used to gather novel evidence on the origin and translational characteristics (i.e., expression levels and accuracy) of specific gene sets (7, 8). Through the employment of 59-variable-based analysis, gene codon compositions have been used to search for genomic heterogeneities (9). Different bacterial taxa carry their own signature codon usages, which contrast with those of the majority of the genes in a genome—those being referred to as the “typical” genes (10, 11).

Gene-by-gene analyses, however, have revealed that, despite this typical codon usage characteristic of each genome, certain gene repertoires manifest a biased form of codon usage that has been related either to their high expression level (12) or to their recent lateral acquisition, for which reason the latter are termed “alien” or “atypical” codon usages (13). For example, highly expressed genes represent a bias in their codon usage that is thought to arise from selection to improve the efficiency and/or accuracy of translation (14, 15). Consistent with this hypothesis, the codon usage of highly expressed genes in different species has been found to correlate well with the tRNA abundances present in the same cells (16). Moreover, distinctive codon usages are present in mobile or accessory genes, an observation that has often been linked to the foreign origin of the genes under analysis (3, 17, 18). Although atypical codon usages had usually been considered the consequence of DNA acquisitions from distant genomic sources (18, 19), such a view has been modified by other authors who suggested that the alien codon usages present in a set of genes were likely acquired from closely related species (17, 20). Why such genes currently present a biased form of codon usage compared to the vertically inherited gene pool within the species is not yet clear. Irrespective of the evolutionary mechanisms that modulate the drift and selection of the codon usages on both the single-gene scale and the genomic scale, certain bacteria have been reported to contain genetic information of plasmid origin bearing a markedly different form of codon usage (9, 21).

In many rhizobial species, the chromosomes coexist with chromids (chromosome-like replicons), megaplasmids, and also smaller accessory plasmids (22). Such multipartite and complex genomes provide suitable models to investigate gene movements within and among bacterial cells, as well as to gain a better understanding of the evolutionary mechanisms that support genome plasticity and functional partition and coevolution among the replicons. In the work reported here, we investigated all these issues using Sinorhizobium meliloti as the bacterial model for the genomic studies. We first sequenced—on a megabase scale—the accessory plasmid DNA (i.e., that of the plasmidome) present and then, together with already available genomic data, analyzed how information is distributed and encoded in the different replicons. The results revealed replicon-associated functional profiles and degrees of variation, together with detectable patterns of codon usage adaptation to the translational machinery, that depended on the gene set under analysis as well as on the location and ancestry of those genes within the rhizobial cell.

## RESULTS

### Deep sequencing of an S. meliloti cryptic plasmidome from a previously characterized collection of strains.

We previously characterized an S. meliloti collection of strains that was built up on the basis of their accessory plasmid diversity (5). With the aim of sequencing the accessory plasmids present in that collection of 18 S. meliloti isolates, their non-pSym plasmids were prepared as described in Materials and Methods. A DNA pool containing an admixture of all the plasmid preparations was sequenced by means of a MiSeq platform, assembled, and filtered to eliminate sequences corresponding to contaminating chromosomal DNA (*cf*. Materials and Methods). The filtered data resulted in 315 contigs (>1,000 bp), which group accounted for a total sequence of 1.46 Mbp of nonredundant plasmid DNA (44 kb for the longest contig, with an average nucleotide sequence length [*N*_50_] of 8.1 kb). The sequences obtained indicated that an average of (at least) 80 kb of accessory plasmid DNA per isolate was present in our collection of strains. The mean level of GC content for the plasmidome was 58.3%, a value that was consistent with the available data for accessory plasmids from several S. meliloti genome projects (https://www.ncbi.nlm.nih.gov/genome/plasmids/1004). While the S. meliloti chromosomes and the pSymB megaplasmids have average levels of GC content of 62.7% and 62.4%, respectively (23, 24), the pSymA megaplasmids have an average level of GC content of 60.4% (25) which is the closer value to the one observed here for the accessory plasmidome. Gene prediction led to the identification of 1,541 putative coding sequences.

### COG class abundances and compositional variations in the S. meliloti plasmidome compared to those present in the pSyms and in the chromosome.

**FIG 1:**
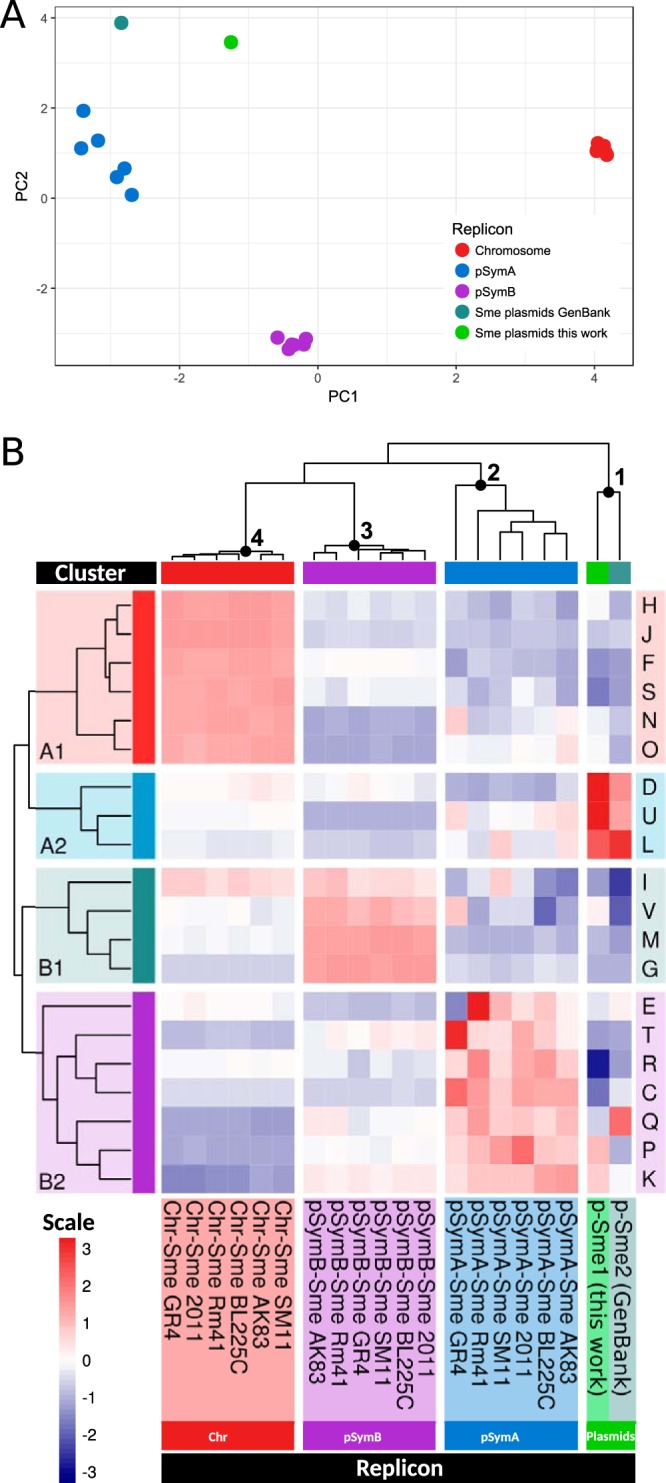
S. meliloti replicon analysis based on the type and proportion of functions encoded therein. (A) PCA-based separation of genes present in the S. meliloti chromosome, pSymA, pSymB, and the cryptic plasmidome according to the differences in the contents of the COGs of those genomic compartments. The color code for the replicons is indicated in the figure. The PCA calculations were performed with the COG proportions in each of the gene sets analyzed as variables (see Materials and Methods). The gene sets were extracted from the complete genomic sequences of S. meliloti strains BL225C (NC_017322.1, NC_017324.1, and NC_017323.1), AK83 (NC_015590.1, NC_015596.1, and NC_015591.1), SM11 (NC_017325.1, NC_017327.1, and NC_017326.1), Rm41 (NC_018700.1, NC_018683.1, and NC_018701.1), GR4 (NC_019845.2, NC_019846.2, and NC_019847.2), and 2011 (NC_020528.1, NC_020527.1, and NC_020560.1). Except for the cryptic plasmidomes, each dot corresponds to a different gene set in a given S. meliloti strain, with “Sme” standing for S. meliloti. (B) Cluster display of the different COG abundances in the genomic locations indicated below the figure. Red, chromosomes (Chr); violet and blue, megaplasmids; green, plasmidome. Each COG type is represented by a single row distinguished by letters on the right side of the figure, while each gene set in a given genomic location and strain is represented by a single column above the corresponding strain identifier within each replicon below the figure. The dendrograms and colors were generated by the software cited in Materials and Methods. The color scale illustrates the change in the proportion of each specific COG among the 20 listed with respect to the average value over the different gene sets. The color scale ranges from white for the average value up to deep red for the highest value and down to deep violet for the lowest. The functions associated with the COG cluster groups (the rows included in those denoted as A1, A2, B1, and B2), designated in the far-left column of the figure, are described in the text.

In order to estimate and compare the relative abundances of cellular functions in the different S. meliloti replicons, the proportion of each class among the Clusters of Orthologous Groups (COG) (classes A to Z) (Fig. 1B) was calculated for the accessory plasmidome reported here and for the chromosomes and pSyms of each of six different S. meliloti strains distributed throughout the phylogeny of the species. The COG proportions calculated for each genomic replicon and for the plasmidome were then used as input variables to perform the principal-component analysis (PCA) whose results are presented in Fig. 1A (from the data in Table S2 in the supplemental material). The results demonstrated that the different replicons—chromosomes, pSymAs, pSymBs, and the plasmidome (indicated as “Sme” plasmids in the figure)—all mapped and clustered in clearly separate regions within the two-dimensional space defined by principal components PC1 and PC2, with these components together representing more than 67% of the total variation. The higher level of functional variation among the pSymAs than among both the pSymBs and the chromosomes is reflected by the broader dispersion of the pSymAs among the strains in the PCA (Fig. 1A, blue dots). The pSymBs (violet dots), frequently referred to as “chromids” (26), exhibited narrower compositional dispersion in the represented COG classes that was comparable to the degree of dispersion observed for the COG classes of the S. meliloti chromosomes (red spheres). This analysis of COG abundances served to generate a description of the degree of functional variation among the S. meliloti replicons. A multivariate hierarchical cluster analysis exploring the distribution of COGs revealed a predominance of specific functional classes associated with each type of replicon (Fig. 1B), with the COGs from cluster A1 (housekeeping functions and motility), cluster B1 (lipid metabolism; defense mechanisms; carbohydrate metabolism and transport; and cell wall, membrane, and envelope biogenesis), and cluster B2 (amino acid metabolism, inorganic ion transport and metabolism, signal transduction, and energy production and conversion) being predominant in the chromosomes, pSymBs, and pSymAs, respectively (as indicated by the predominance of red hues in those areas of the figure). The S. meliloti plasmidome emerged as a separate genomic component group with a dominance of activities associated with trafficking and secretion (COG class U) and with functions—as expected—related to plasmid maintenance and stability (COG classes D and L). Finally, a more highly conserved form of COG composition was observed both in the chromosomes and in the pSymBs than was seen with the COG class heterogeneity among the pSymAs—with this variation being visualized in Fig. 1B as a range of red hues for a given COG class represented in each of the pSymA columns. That the pSymBs had grouped with the chromosomes, according to the functional profile, was remarkable (clades 3 and 4 indicated above the dendrogram of Fig. 1B). In agreement with the PCA data and with the color profiles in the heat map, pSymA was found to be the replicon that was most closely related to the plasmidome in terms of the encoded functions as deduced from the COG distances presented in Table S2 (“COG distances” tab).

10.1128/mBio.00505-19.4 TABLE S2 Functional description of the different Sinorhizobium meliloti replicons in terms of the proportion of proteins that belong to each of the Clusters of Orthologous Groups (COGs). Functional-COG distances among replicons are indicated. Download Table S2, XLSX file, 0.04 MB. Copyright © 2019 López et al. 2019 López et al. This content is distributed under the terms of the Creative Commons Attribution 4.0 International license.

### Evidence for gene transfer between the S. meliloti accessory plasmidome and the other rhizobial replicons: the GC content and codon usage in different gene sets.

**FIG 2:**
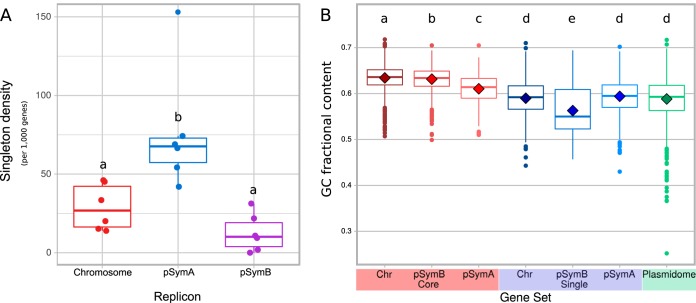
Density of singletons in the three S. meliloti replicons (A) and GC content of their core and singleton fractions in comparison to that of the cryptic plasmidome (B). In the box plots shown in panel A, the average numbers of singletons per 1,000 genes are plotted on the ordinate for the S. meliloti chromosome, pSymA, and pSymB as indicated on the abscissa. The color codes are as follows: chromosome, red; pSymA, blue; pSymB, violet. The median values presented in the figure were calculated using the genes of the same six S. meliloti strains as are listed in the Fig. 1A legend. The lower and upper hinges correspond to the first and third quartiles (the 25th and 75th percentiles). The upper whisker extends from the hinge to the largest value no further than 1.5× the IQR from the hinge (where “IQR” is the interquartile range, or the distance between the first and third quartiles). The lower whisker extends from the hinge to the smallest value (at most, 1.5× the IQR of the hinge). Data beyond the end of the whiskers are “outlying” points and are plotted individually. Panel B contains box plots depicting, as indicated on the ordinate, median GC content values (solid lines), with diamonds indicating the mean values for the designated gene sets—i.e., the core group and the singletons of the chromosome and the pSyms along with the cryptic plasmidome, as denoted on the abscissa. In both panels, the data corresponding to the gene sets indicated by the same letters were not significantly different according to the pairwise Wilcoxon test for multiple comparisons performed with the R package (*P* = 0.05).

**FIG 3:**
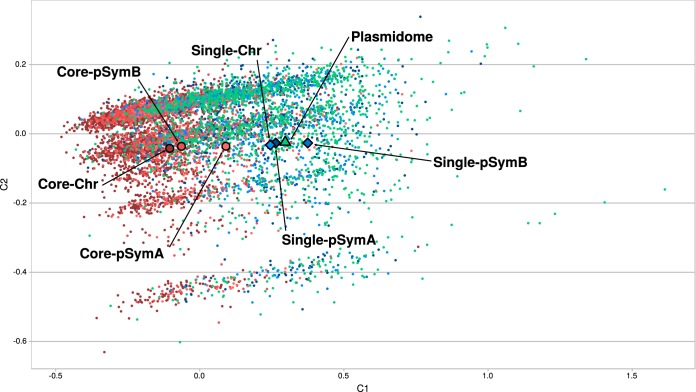
Factorial correspondence analysis-based separation of different S. meliloti gene sets (cores and singletons) according to their different codon usage profiles. Individual genes and gene sets (observations) were separated in the correspondence analysis (CA) plot on the basis of the proportion of each of the 59 codons that had synonymous triplets (variables). The relative synonymous codon usage (RSCU) values for individual genes—calculated as the observed frequency of a given codon divided by the frequency expected under the assumption of equal levels of usage of the synonymous codons for an amino acid (56)—and the modal codon usages (for the gene sets) employed in the CA were calculated by the use of CodonW software (J. F. Peden, http://codonw.sourceforge.net/) and through an application of the algorithm of Davis and Olsen (9), respectively. The positions of individual core and singleton genes from the chromosome, pSymA, and pSymB are indicated by small dots in the space corresponding to the first two components of the CA (i.e., C1 and C2) in the figure and in accordance with the following color codes: red, variants among the core genes; blue, variants for singletons. The genes from the S. meliloti cryptic-plasmid mobilome are indicated by small green dots. The position of the modal codon usage value of each gene set is denoted through the use of larger symbols (respective dark-red to light-red circles for the chromosomal, pSymA, and pSymB core genes; dark-blue to light-blue diamonds for the chromosomal, pSymA, and pSymB singletons; and a sole green triangle for the plasmidome).

The analysis described in the previous section demonstrated that the different genomic components in S. meliloti bear—with diverse degrees of variation—distinctive functional profiles. We next investigated the possible origin and sources of the observed gene content variations within the same type of replicon. Panel A of Fig. 2 indicates the average number of singletons (unique, exclusive genes) per thousand genes in the S. meliloti chromosome, pSymA, and pSymB. Consistent with the COG class variations found among the pSymAs (Fig. 1), these replicons also contained the highest density of singletons. In the Bacillus cereus group, chromosomal singletons were considered to represent the more recently acquired genes from the mobile plasmid pool (21). The data in Fig. 2A may suggest that horizontally acquired genes are likely to be those most frequently incorporated into the pSymAs (thus representing a “hot sink”) followed by the chromosomes and, finally, the pSymBs, though the densities in the latter two compartments were quite similar (24). Two pieces of evidence point to the accessory plasmidome as a major source of the observed S. meliloti replicon singletons. First, the average GC content calculated for the S. meliloti accessory plasmidome was fairly similar to that calculated for the singletons but differed from the GC content of the core genes irrespective of the replicon under analysis (Fig. 2B). Second, the correspondence analysis (CA) data presented in Fig. 3 indicated that the modal codon usage of the singleton fractions of all the genomic components (diamonds) was close to that observed for the accessory plasmidome (green triangle). The CA plot demonstrated that these close relative locations contrasted with the more distantly located positions of the core fractions (circles) and the plasmidome. Table 1 lists the numerical distances between the data representing the modal codon usages of specific gene sets. In agreement with the results presented in the CA plot, the codon usage distances from the plasmidome to each core fraction (0.326 to pSymA, 0.575 to pSymB, and 0.633 to the chromosome) were significantly greater (at a 95% confidence interval according to the Mann-Whitney test; *cf*. Materials and Methods) than the distances from the plasmidome to each singleton fraction of the corresponding replicon (i.e., 0.110 to pSymA, 0.194 to pSymB, and 0.143 to the chromosome). In addition to the clear CA relatedness of the singletons and the plasmidome genes, the singletons of those first three genomic component groups all comprised levels of modal codon usages that are significantly different from those observed for the plasmidome (Mann-Whitney test [95% confidence interval]).

**TABLE 1:** Computed modal codon usage distances among selected gene sets

Gene set 1^*a*^	Gene set 2	Distance between gene set modes	Mean distance after bootstrap resamplings of the gene sets ± SD^*b*^		Mean distance between shuffled gene sets ± SD^*c*^	
CA	P	0.325	0.326	0.019	0.049	0.010
SA	P	0.100	0.110	0.018	0.045	0.009
CB	P	0.572	0.575	0.014	0.049	0.011
SB	P	0.176	0.194	0.029	0.082	0.015
CC1	P	0.628	0.633	0.013	0.038	0.007
SC	P	0.129	0.143	0.014	0.047	0.008
CC1	CA	0.31	0.319	0.017	0.044	0.008
CC1	CB	0.089	0.098	0.009	0.035	0.006
CB	SB	0.695	0.689	0.051	0.067	0.011
CA	SA	0.238	0.243	0.024	0.053	0.010
CC1	SC	0.571	0.575	0.017	0.042	0.007
SA	SB	0.239	0.247	0.044	0.083	0.017
SA	SC	0.087	0.108	0.012	0.053	0.009
SB	SC	0.214	0.230	0.042	0.090	0.016
CC1	CC2	0.027	0.047	0.008	0.036	0.006
CC1	CC3	0.030	0.050	0.008	0.037	0.006
CC1	CC4	0.063	0.075	0.008	0.038	0.007
CC1	CC5	0.080	0.090	0.008	0.039	0.007
CC1	CC6	0.083	0.094	0.009	0.040	0.007
CC1	CC8	0.103	0.112	0.01	0.042	0.008
CC1	CC9	0.117	0.125	0.011	0.044	0.008
CC1	CC10	0.118	0.129	0.012	0.045	0.008
CC1	CC12	0.142	0.149	0.012	0.047	0.008
PHEL	CC15	0.106	0.161	0.019	0.101	0.017

a Gene set abbreviations are as follows: CC1, chromosomal core; CC*n*, chromosomal cores of increasing ancestry; CB, pSymB core; CA, pSymA core; SA, pSymA singletons; SB, pSymB singletons; P, S. meliloti plasmidome; PHELs, protein species with the highest expression levels in S. meliloti.

b The bootstrapped means and SDs were calculated from 1,000 replicates.

c The means ± SDs correspond to 1,000 resampled replicates.

An analysis of the chromosomal and megaplasmid core genes enabled an investigation of the codon usage profiles within the most conserved genomic replicons in S. meliloti. The decay function of the core development plot seen under conditions of an increasing number of pSyms (see Fig. S1A and B in the supplemental material) suggested that precursor plasmids of pSymA and pSymB in the S. meliloti phylogeny would likely have had core sizes of no more than 200 and 1,100 genes, respectively, constituting 20% and 80% of the current total gene content in the corresponding replicons. This observation reinforced the notion of the highly plastic character of the pSymAs. The CA data presented in Fig. 3 and Table 1 illustrate that the core fractions of the different replicons evidenced modal codon usages that were distinguishable among the three, with the modal codon usage of the pSymA core fraction being the closest to that of the plasmidome. Furthermore, the distance between the core and singleton fractions in pSymA (0.242 ± 0.024; see CA and SA data in Table 1) was less than the corresponding distance in pSymB (0.689 ± 0.051; see CB and SB data in Table 1) and in the chromosome (0.575 ± 0.017; see chromosomal core gene set 1 [CC1] and synonymous codon [SC] data in Table 1). The pSymB and chromosomal core genes were likewise close to each other in the CA plot (0.098 ± 0.009; see CC1 and CB data in Table 1), with the respective distances to the singletons—i.e., the CB and SB singletons and the CC1 and SC singletons in Table 1—and to the plasmidome fractions—i.e., CB or CC1 and P in Table 1—being the greatest. These results, taken together, indicated that the more highly conserved (i.e., ancestral and thus adapted to the host) a gene set in S. meliloti was, the more distant its modal codon usage was from that of the plasmidome.

10.1128/mBio.00505-19.1 FIG S1 Decay functions of the number of core genes in the S. meliloti pSym replicons with respect to an increase in the number of strains under analysis. The decay functions were adjusted to fit a nonlinear, least-squares model by the use of EDGAR software. The upper and lower confidence intervals are delimited by gray dashed lines. (A) pSymA. (B) pSymB. In each panel of the figure, the number of core genes per replicon is plotted on the ordinate as a function of the number of genomes under consideration. Download FIG S1, PDF file, 0.1 MB. Copyright © 2019 López et al. 2019 López et al. This content is distributed under the terms of the Creative Commons Attribution 4.0 International license.

### A progressive analysis of codon usage in different and sequential core fractions throughout rhizobial phylogeny.

**FIG 4:**
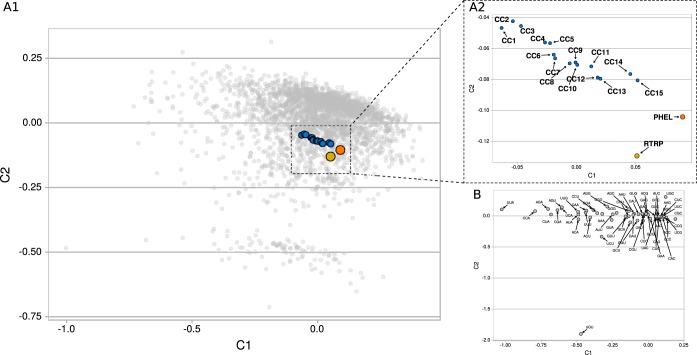
CA-based separation of sets of S. meliloti chromosomal core genes with different degrees of conservation throughout rhizobial phylogeny with respect to the corresponding codon usage profiles of those sets. (A1) The position of the modal codon usage of different S. meliloti subsets of core genes is indicated in the space of the first two components of the CA with blue dots, representing numbered chromosomal core genes (CCs; *cf.* panel A2), where the numbers increase with progressively deeper retrospective penetration into the proteobacterial phylogeny. Table S5 lists the bacterial species that were used to construct each S. meliloti CC by means of EDGAR software (45). (A2) The modal codon usages for the S. meliloti genes that correspond to proteins with the highest expression levels (PHEL) and to ribosomal and other translation-related proteins (RTRPs) are labeled accordingly. A magnified view of the boxed region in panel A1 displays the positions of modal codon usages for the indicated gene sets. (B) A loading plot describing which codons have the most pronounced effect on the first two principal components of the CA presented in panel A. Variations in the use of synonymous codons have a stronger effect on component C1, with that axis being directly related to ancestry. The more ancestral the core fractions were, the farther to the right they became located; and the more frequent was the use of synonymous codons having G or C at the third position (see, e.g., the codons on the right side of the plot; *cf*. also with Fig. 7C for the evolution of the average GC content at the third position of the codons).

**FIG 5:**
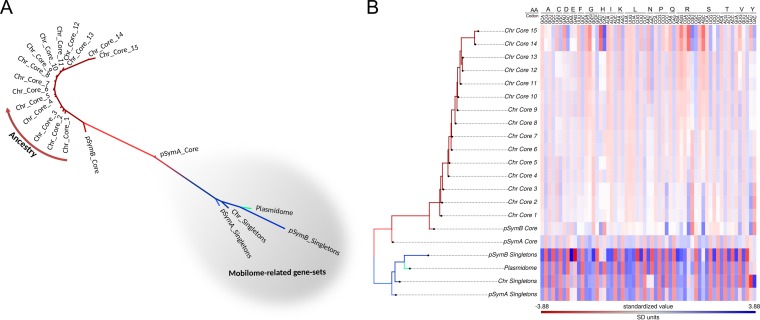
Neighbor-joining distance tree (A) and heat map (B) of different S. meliloti gene sets based on their modal codon usage. (A) The modal codon usage data corresponding to different S. meliloti genes sets were used to construct a neighbor-joining tree according to the method described by Karberg et al. (20) along with the neighbor program of the Phylip package (46). Phylogenetic trees were visualized and edited by the use of the Figtree application (47). Tree branches are colored (here and in panel B) as follows: chromosomal core genes with increasing ancestry, dark red; pSymB core genes, medium red; pSymA core genes, light red; chromosomal singletons, dark blue; pSymB singletons, medium blue; pSymA singletons, light blue; plasmidome, light green. Gene sets that belong to the mobilome of the species are highlighted with a gray background. (B) The modal codon usages of the indicated gene sets were used to construct a heat map with the rows ordered by the same neighbor-joining tree as described for panel A. The heat map was constructed using the phytools R package (57). The color scale from red to blue indicates the relative level of change in each codon modal codon usage (column). Amino acids are indicated by the use of one-letter codes.

The CA in Fig. 3 revealed a directional shift in the modal codon usage of the core genes with respect to that of the singletons in the same replicon (i.e., a displacement from right to left in the first component in the CA). On the basis of this observation, we examined relationships between the core-gene ancestry and codon usage. Thus, different sets of S. meliloti chromosomal core genes (*Sme*-CC1 to *Sme*-CC15) were reconstructed, as such progressing deeper into the rhizobial phylogeny (see Table S5 and the embedded figure). The modal codon usages of the different S. meliloti CCs were calculated and incorporated into a comprehensive CA (see panels A1 and A2 of Fig. 4). The results demonstrated that the positions of the modal codon usages were sequentially ordered, with the distances to the CC1 increasing in accordance with the CCi gene sets containing more concentrated ancestral orthologs within the lineage. Table 1 documents how the distance from CC1 to CC*n* progressively increases with increasing “*n*” values. The core gene sets presented a pattern of codon usage adaptation that paralleled ancestry (i.e., from CC1 to CC15). The relative positions among the core fractions, singletons, and plasmidome are summarized in the neighbor-joining tree inferred from codon usage distances that is presented in Fig. 5A. The results show (i) all core fractions located sequentially on one side of the tree; (ii) all components related to the mobilome (i.e., plasmidome and singletons) grouping together and distant from the core genes on the other side of the tree, and (iii) the pSymA core genes mapping at an intermediate position between the more ancestral core genes (chromosome and pSymB) and the mobilome. The heat map (Fig. 5B) depicts which particular synonymous codons characterize each gene set. Strong enrichment in many C-ending codons (more intensely blue colors) is evident in several amino acids of CC14 and CC15 (e.g., in A, C, D, F, G, H, I, L, N, R, S, T, V, and Y) in comparisons of those fractions to the plasmidome and singletons. As with the previously described C bias, a specific enrichment in several G-ending codons (e.g., in L, P, K, Q, S, and T) was also observed in the more ancestral core genes.

10.1128/mBio.00505-19.7 TABLE S5 Bacterial species used in this work for the calculation of the S. meliloti core gene sets CC1 to CC15. Download Table S5, XLSX file, 0.4 MB. Copyright © 2019 López et al. 2019 López et al. This content is distributed under the terms of the Creative Commons Attribution 4.0 International license.

**FIG 6:**
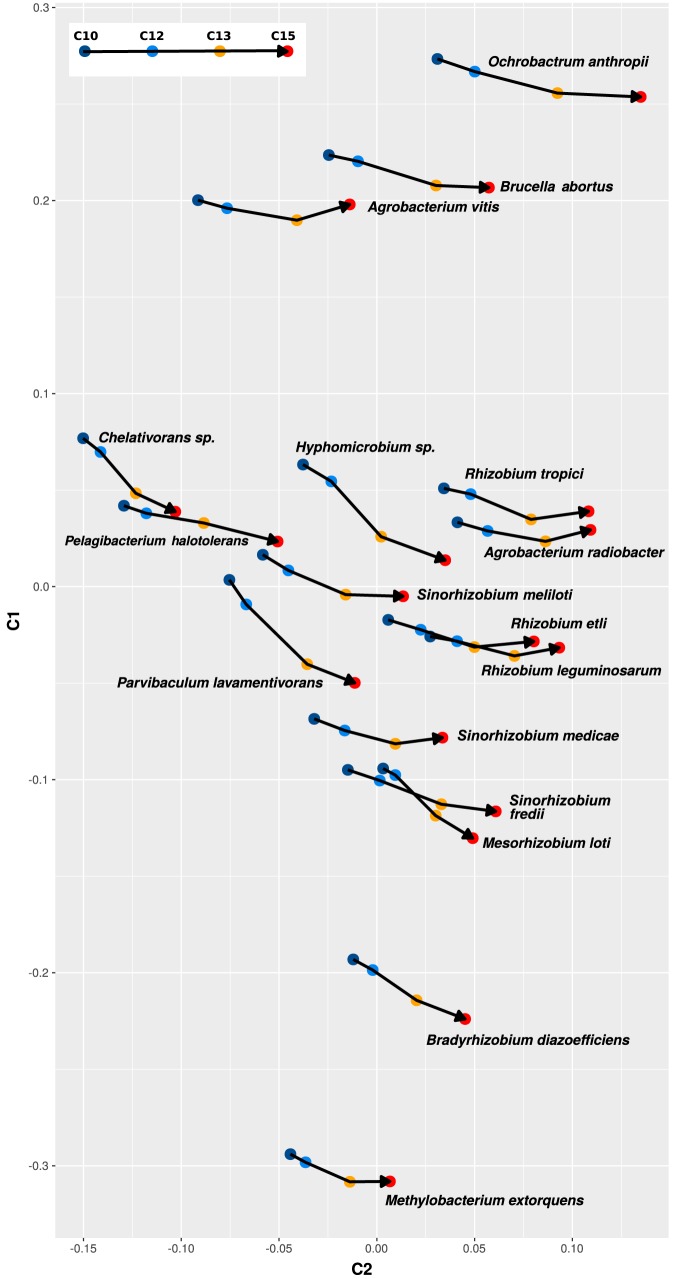
CA-based separation of chromosomal core sets of genes with increasing ancestry from the indicated bacterial genomes according to the differences in codon usage. The modal codon usages corresponding to different subsets of chromosomal core genes with increasing ancestry for a given bacterial species are connected by lines. Modal values correspond to genes of chromosomal core sets CC10 (dark blue), CC12 (light blue), CC13 (orange), and CC15 (red) as indicated in Table S5. The modal codon usages were calculated as previously described by Davis and Olsen (9). C1 and C2 in the ordinate and abscissa correspond to principal components 1 and 2, respectively. The arrowheads indicate the direction of ancestry.

**FIG 7:**
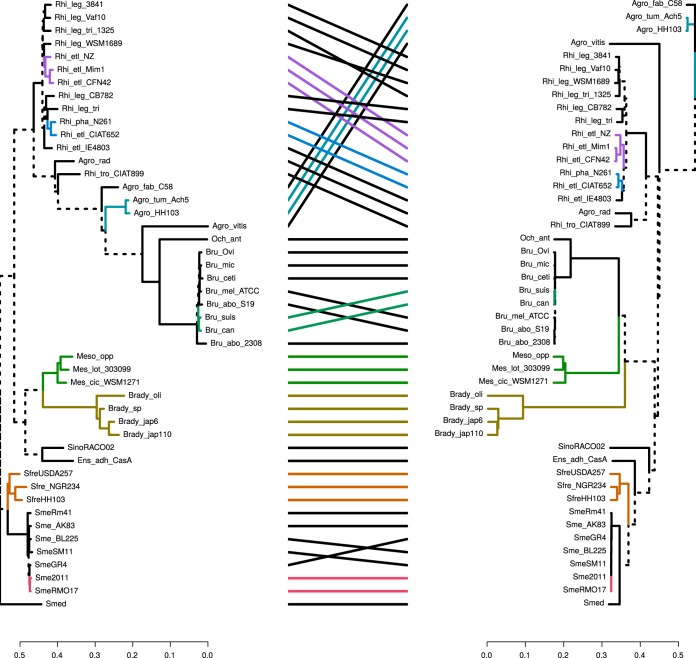
Phylogenetic relationships among selected members of the order *Rhizobiales* based on the comparative analysis of either the codon usages of the corresponding structural genes (left panel) or the amino acid sequences of chromosomally encoded core proteins (right panel). (Left panel) The phylogenetic tree was generated on the basis of the modal codon usage distances among all the indicated species, with the modal values calculated for the gene sets that corresponded to the proteins used in the right panel. The modal codon usages of each set of orthologous genes were calculated as previously described by Davis and Olsen (9). The phylogenetic tree was generated according to a method previously described by Karberg et al. (20). (Right panel) The phylogenetic tree was generated with EDGAR software (45) on the basis of the translated amino acid sequences of 725 chromosomal core genes present in the 48 bacterial species listed in Table S6. (Central panel) A tanglegram was constructed to compare the two trees using the APE package (58) and dendextend (59) R package. Unique nodes (i.e., nodes containing a combination of labels not present in the other tree) are highlighted with dashed lines. Connecting lines are colored to highlight subtrees (also colored) which are present in both dendrograms. The Ape R package was used to compute the normalized Robinson-Foulds distance between the phylogenetic trees depicted in panels A and B, resulting in a value of 0.57 (i.e., 43% branch coincidence between both trees).

A broader inspection of this phenomenon (Fig. 6) revealed that a species-specific positional shift of the codon usage in the core genes also became evident in other species of the order *Rhizobiales*. Thus emerged the issue of to what extent the codon usages reflected the taxonomic positions among the different bacterial taxa. Neighbor-joining trees that explore the congruence between the amino acid phylogeny and the codon usage phylogeny of different rhizobia and related bacteria are shown in Fig. 7. Results show an exact correspondence in ca. 40% of the splits according to the normalized Robinson-Foulds distance (APE package in R; see Materials and Methods). Nearly all the observed correspondences mapped, as expected, at the species level (see central tanglegram in Fig. 7). The congruence between the two trees rapidly decreased at higher taxonomic ranks, preserving comparable clusters in only some species’ groups (i.e., S. meliloti-S. medicae-S. fredii and Rhizobium etli-R. leguminosarum-R. phaseoli) and genera (i.e., *Mesorhizobium*-*Bradyrhizobium* and *Brucella*-*Ochrobactrum*). The observation reflects the fact that strong species-specific codon usage adaptations differentiate even phylogenetically closely related bacteria.

10.1128/mBio.00505-19.8 TABLE S6 Accession numbers used for the construction of the amino acid-based phylogenetic tree presented in Fig. 7. Download Table S6, XLSX file, 0.01 MB. Copyright © 2019 López et al. 2019 López et al. This content is distributed under the terms of the Creative Commons Attribution 4.0 International license.

### Codon usage adaptation of core genes, tRNA abundance, and protein expression levels.

**FIG 8:**
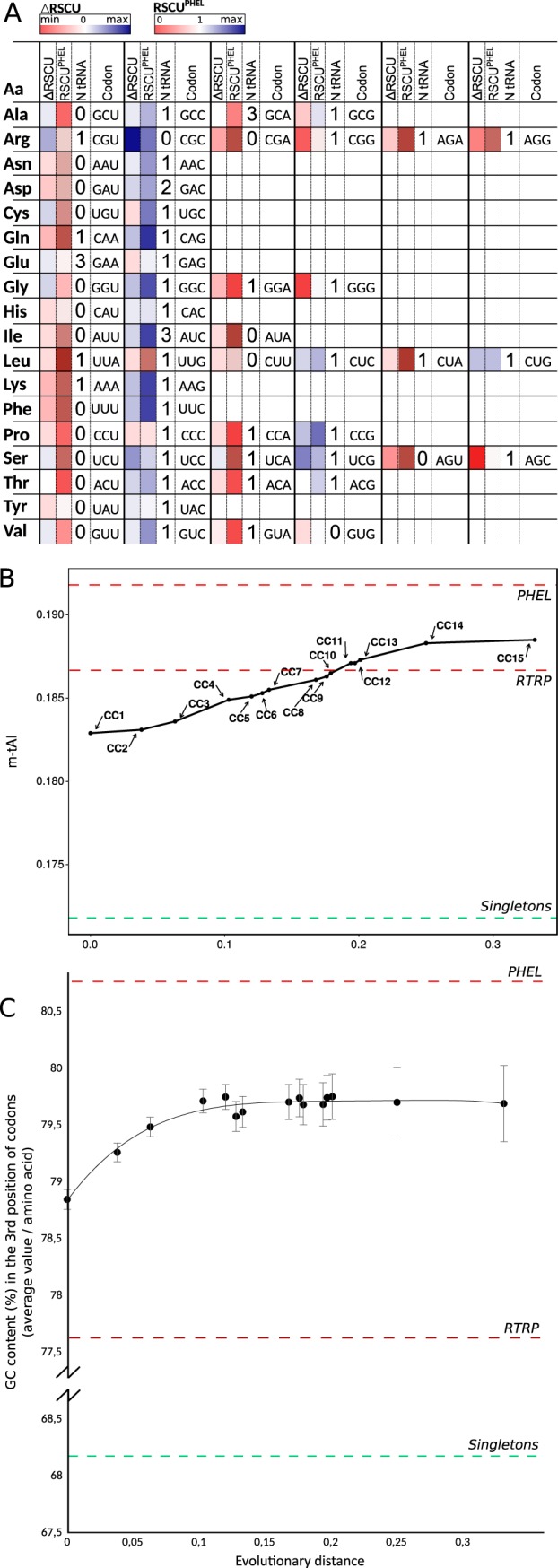
Ancestry-dependent codon usage adaptation of S. meliloti chromosomal core genes (CCs) to the tRNA gene set and changes in the GC3 content. (A) Change in the abundance of each codon (enrichment) in core-gene sets with increasing ancestries (CC1 to. CC15). After computing the relative synonymous codon usages (RSCUs) through general codon usage analysis (GCUA) (60), the enrichment in a given codon “i” was calculated as [_CODON “i”_RSCU^CC15^ − _CODON“i”_RSCU^CC1^]. In the rectangles corresponding to the ΔRSCU of each entry, the more intense the blue color is, the more enriched is the corresponding codon in the CC15 gene set than in the CC1 gene set, while the more intense the red color is, the more enriched is the corresponding codon in the CC1 than in the CC15. The boxes in white indicate equal abundances (RSCUs) of the designated codons in the two core-gene sets. The rectangles corresponding to the RSCU^PHEL^ indicate in color scale—as a reference corresponding to the more highly adapted genes—the RSCU for each codon in genes that correspond to PHELs. The numbers on the left side of each codon indicate the copy number of the corresponding tRNAs recognized by Watson-and-Crick base pairing. (B) The mean tRNA adaptation indices (m-tAIs) calculated for the different numbered CC gene sets as indicated in Materials and Methods (with the higher number corresponding to the more ancestral core gene set) are plotted on the ordinate as a function of the evolutionary distances on the abscissa. Higher codon usage frequencies in those synonymous codons that improve the interaction with the available tRNA pool result in higher values of m-tAI. The more relevant parameters affecting the m-tAI values are the tRNA copy number and the selective constraints related to Crick’s wobble rules. The evolutionary distances on the abscissa correspond to those inferred from the phylogeny tree of Table S5. The CCs here are the same as the chromosomal core gene sets presented in Fig. 4. The horizontal dashed lines in different colors correspond to the calculated m-tAI values for the experimentally determined PHEL, RTRP, and singletons. (C) Evolution of the average GC content at the third position of codons (GC3) in different sets of core genes with increasing ancestry. In the figure, the average percent GC3 for several sets of core genes is plotted on the ordinate as a function of ancestry on the abscissa using the same evolutionary distances as those described for panel B. The vertical bars represent the standard deviations (SDs). The red and green dashed lines in panels B and C correspond to the calculated values for the experimentally determined PHELs and for the singletons, respectively.

In several organisms, including S. meliloti, highly translated products such as several ribosomal and other translation-related proteins (RTRPs) have been reported to exhibit codon usages that are significantly different from that corresponding to the complete genome (27, 28). In the present study, we obtained an experimental proteome for S. meliloti growing in defined medium (Materials and Methods), which characterization served to identify those protein species with the highest expression levels (PHELs) under the conditions analyzed. As had been previously found for RTRPs in rhizobia and other organisms (29, 30), the preferred codons encoding those experimentally observed PHELs in S. meliloti correlated positively with a higher abundance of the tRNAs that were able to recognize those codons through exact Watson-and-Crick hydrogen-bonding interactions (Fig. 8A). In the table shown in Fig. 8A, the codons with the darker blue color in most instances had a cognate tRNA present in the cell, with that species often being the one with the highest copy number.

A similar adaptation to the translational machinery was also observed in comparing RSCUs from core genes of CC1 with those of CC15 (Fig. 8A, colors on the left side of the boxes). Thus, codon usage adaptation to the tRNA pool was not restricted to the genes encoding PHELs. No harmonization occurred between the more abundant tRNAs (in terms of copy number) and their cognate codons in only 3 (Ala, Arg, Cys) of the 18 amino acids encoded by more than one codon. The adaptation between the codon usage of the chromosomal core genes and the tRNA pool is reflected in the progressive increase in their modal tRNA adaptation index (m-tAI) values plotted against the evolutionary distance (Fig. 8B). Comparable results were obtained for the codon usage optimization estimated through the codon adaptation index (CAI) as suggested by the data reported in reference 31 (*cf.* Table S7 data). The results indicated that an increase in the levels of those codons recognized by the more abundant tRNA anticodons correlated well with gene ancestry, an adaptation that in S. meliloti was also accompanied by an average GC increase in the third position of the codons (Fig. 8C). A similar analysis of other related bacteria (Fig. S2) revealed that the observed increase in the m-tAI value with core-gene ancestry is part of a more general phenomenon that is operative in several other species of the order *Rhizobiales*. Such observations indicate that the coevolution of codons and tRNAs takes place on the genomic scale in bacteria, in addition to the already thoroughly documented adaptation of the quantitatively more highly represented protein species such as RTRPs and PHELs.

10.1128/mBio.00505-19.2 FIG S2 Ancestry-dependent codon usage adaptation of chromosomal core genes (CC*n*) from bacterial species of the order *Rhizobiales* to the corresponding tRNA gene set. In each of panels a through q, the tRNA adaptation index (t-AI) values are plotted on the ordinate for each of the sets of chromosomal core genes (CCs) listed on the abscissa. As indicated in the legend to Fig. 7, the estimation of the translational efficiency shown on the ordinate corresponds to the m-tAI values calculated for CC10 to CC15 (increasing ancestry) as those values are defined in Table S5. The bars correspond to the m-tAI values calculated for the ribosomal and other translation-related proteins (RTRPs). Download FIG S2, PDF file, 0.2 MB. Copyright © 2019 López et al. 2019 López et al. This content is distributed under the terms of the Creative Commons Attribution 4.0 International license.

10.1128/mBio.00505-19.9 TABLE S7 Gene copy number of all tRNA species in S. meliloti 2011 and in the other bacteria used in this work. Download Table S7, XLSX file, 0.02 MB. Copyright © 2019 López et al. 2019 López et al. This content is distributed under the terms of the Creative Commons Attribution 4.0 International license.

## DISCUSSION

The symbiosis model soil bacterium S. meliloti bears a multipartite genome composed of a chromosome; a highly conserved megaplasmid (i.e., pSymB, also referred to as a “chromid”); a second but nonessential megaplasmid (i.e., pSymA); and, in many strains, one or more accessory plasmids as well—with sizes ranging from a few to hundreds of kilobases—that are mostly still cryptic with respect to knowledge of their function (4, 5, 23–25). That genomic structure in S. meliloti—together with the availability of data on the genomes of this and other related rhizobial species—provides a most suitable system to investigate structural, functional, and evolutionary issues in a model bacterial genome. In addition to the well-documented evidence supporting a basic structural conservation of chromosomes, pSymBs, and pSymAs within the S. meliloti species (32–34), the analysis presented in Fig. 1—based on a comparison of the COG-content profiles—clearly demonstrated a higher degree of variation among the pSymAs, with their functional profiles being closer to that of the cryptic plasmidome than to those of the chromosomes or the pSymBs—an observation consistent with a recent report by Nelson et al. (34). While the pSymAs are enriched in genes associated with signal transduction (COG-T) and energy production (COG-C) compared to the plasmidome, the latter is exceptionally enriched in genes promoting trafficking and transport (COG-U). Replicon plasticity has been frequently associated with the density of singletons as an expression of recently incorporated genes (21). In agreement with the higher functional variation among pSymAs, those replicons exhibited the highest concentration of singletons and must therefore be the most permissive among the three conserved replicons for the incorporation of novel genes. Moreover, the singleton fractions from the chromosomes, the pSymBs, and the pSymAs all possessed GC contents and manifested codon usages that were comparable to those of the genes that were part of the cryptic plasmidome, thus suggesting that most of those singletons had likely originated in a common mobile gene pool. In contrast to this pattern, all the core fractions—i.e., those including the more highly conserved genes in a given lineage—possessed significantly higher GC contents than the corresponding singletons, irrespective of the replicon under analysis. Thus, the more ancestral (core) genes in S. meliloti are (on the average) the ones with the higher GC content. Nevertheless, that the core fraction of pSymAs had evidenced a separate and lower level of GC content than did the other core fractions was remarkable and suggested that pSymA core genes might well experience selection-adaptation processes more similar to those that operate on the cryptic plasmids than to those that affect the core fractions in the pSymB and the chromosome. In agreement with these considerations, the CA of codon usages illustrated in Fig. 3 also indicated a separate modal value for the pSymA core fraction that not only mapped apart from those of the chromosome and the pSymB but also approached the value corresponding to the modal codon usage of the plasmidome.

The differences observed in the S. meliloti core genes led us to explore in more detail the adaptational changes in the modal codon usages within the chromosomal core genes that were sequentially more ancestral in the lineages of the species (Fig. 4; see also Fig. 5A). The results pointed to a directional shift in the codon usage of the core genes that correlated with the S. meliloti gene ancestry. The adaptation to the translational machinery of CC14 and CC15 compared to the plasmidome and singletons was evident in the heat map presented in Fig. 5B, where a strong increase in C-ending and G-ending codons occurred in several amino acids, in agreement with previous reports in this and in other bacterial species (28, 35). Such adaptation is responsible for the GC3 change and the m-tAI increase that characterized the chromosomal core series CC1 to CC15 (Fig. 8B and C). What was noteworthy was that the more ancestral was the core under consideration, the closer was the corresponding codon usage relative to that of the gene set with the highest expression in the rhizobia (i.e., the PHELs). Codon usage in highly expressed genes has been reported to correlate well with the composition of the cellular tRNA pool in eubacterial and multicellular organisms (27, 36) as well as in certain archaea (37). In S. meliloti in particular, codon usage has also been reported as a main force shaping translational speed for the transcripts of genes in the chromosome and in pSymB (28). We ourselves observed here that the more ancestral were the chromosomal core genes, the better adapted was their codon usage to the S. meliloti tRNA pool (Fig. 8A and B). Codon usage adaptation in S. meliloti occurred in core-protein genes with diverse expression levels. Those changes in the modal codon usage likely operated to improve bacterial fitness through the use of codons that were better adapted to the translational machinery. We have not evaluated whether the bias in the core-gene codon usage over the ancestry of the S. meliloti genes was associated with changes in translational speed and/or accuracy such as had been previously demonstrated for the members of the family Enterobacteriaceae (38). The codon usage adaptation of the core genes was also observed when we excluded the RTRPs from the analysis (not shown). An improvement in fitness had most likely occurred through an increase in the translation efficiency (speed/accuracy) of many (chromosomal core) protein species of diverse expression levels that together represented a significant proportion of the cellular biomass. That shifts in the codon usage of core genes together with a concomitant translational adaptation had been operative in different *Rhizobiales* species (*cf.* the m-tAI values in Fig. S2 in the supplemental material) reflected an extended phenomenon of coevolution in alphaproteobacteria through which process codons and tRNAs were progressively (co)selected to (quantitatively) accommodate their compatibility.

**FIG 9:**
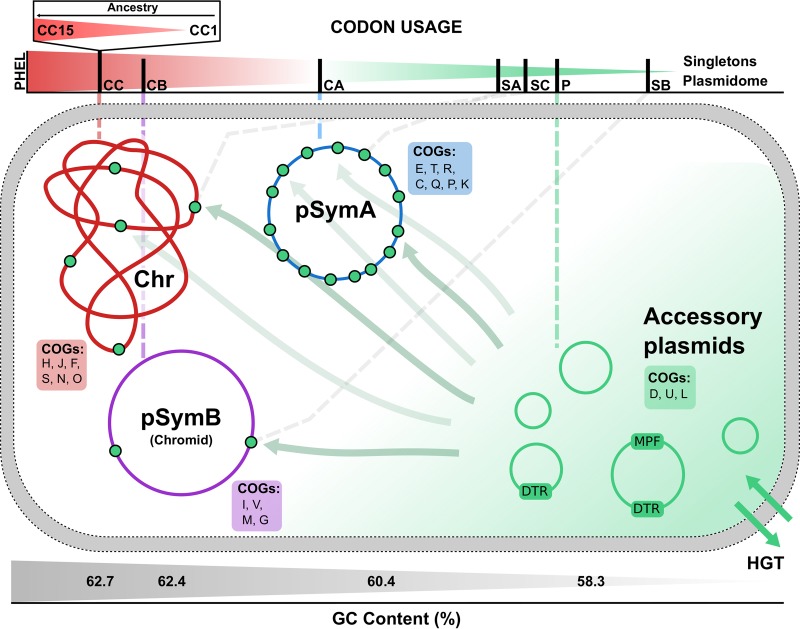
Schematic summary featuring the presence of singletons in the different replicons, the more abundant COGs in each genomic location, and a continuous representation of the modal codon usage in the different S. meliloti gene sets described in this work. The plasmidome and the singletons are indicated in green, with the highest density outside the plasmidome being in pSymA. The most abundant COGs in each replicon and in the plasmidome are indicated with capital letters within color-coded boxes (*cf.* Fig. 1B and the corresponding text). A comparison of modal codon usages for different gene sets (designated with two capital letters) is presented in a continuous array from green (plasmidome) to red (PHELs) above the schema. The positions in the colored scale of the different gene sets were estimated through the values of their corresponding first components in the CA plot presented in Fig. 3. As indicated in Table 1, the abbreviations for the gene sets are as follows: CC, chromosomal core genes; CB, pSymB core genes; CA, pSymA core genes; SC, chromosomal singletons; SB, pSymB singletons; SA, pSymA singletons; P, plasmidome genes. The key to CC15, CC1, and PHELs is the same as indicated in Fig. 7. The gray scale below the schema indicates the approximate GC content for the different genomic compartments.

The overall evidence garnered here demonstrated that S. meliloti has a multipartite genome where each replicon has evolved to contain information associated with a specific group of functional profiles in the form of unique (singleton) and shared (core) genes within the species plus codon usages that proved to be diverse with respect to all these characteristics (as depicted in the graphic summary presented in Fig. 9). In particular, the cryptic plasmidome was found to have functional and compositional characteristics—GC3 and codon usages—that were comparable to those present in the singletons. The analyses presented in this work reinforced the notion of a close relationship between the cryptic plasmidome and the new genes that had been incorporated into the different replicons. That codon usage in the plasmidome resembled the average trinucleotide composition of the S. meliloti intergenic regions independently of the replicon under analysis (not shown) suggested that plasmid genes likely undergo frequent frameshift mutations and recombinational changes that modify their codon usage. The predicted suboptimal translational efficiency in the plasmidome, as deduced from that replicon's modal codon usage, is consistent with the need to avoid sudden detrimental effects on the bacterium upon the arrival of hundreds of mobile genes of plasmid origin. We previously demonstrated that the S. meliloti cryptic plasmidome includes many highly mobile plasmids conferred via conjugation (5). The cryptic plasmids in S. meliloti thus emerge as very likely representing the most highly plastic compartment and one where novel genes are created to then be incorporated as new singletons into the more stable replicons. In those genomic compartments, codon usage amelioration, in turn, gradually achieves an accommodation to the translational machinery of the bacterium.

## MATERIALS AND METHODS

### Sinorhizobium meliloti isolates used in this work.

In order to collect data representing the genetic diversity of non-pSym plasmid DNA—i.e., DNA from neither the constitutive megaplasmid pSymA nor the characteristic chromid pSymB—in S. meliloti, we used the following collection of 18 isolates of the species that had been previously characterized by Pistorio et al. (5): LPU5, LPU9, LPU15, LPU26, LPU57, LPU86, LPU87, LPU88, LPU110, LPU116, LPU118, LPU121, LPU122, LPU178, LPU196, LPU243, LPU244, and LPU258. These rhizobia—all with different plasmid profiles—had been recovered from alfalfa root nodules collected from 25 different neutral soil samples with histories of alfalfa cultivation in Argentina. Pistorio et al. (5) reported a total of 38 plasmid bands in the complete collection of isolates (1 to 4 plasmid bands per rhizobium). The reported cumulative Shannon index values calculated by the frequency of appearance of the different plasmid profiles had approached a plateau, indicating that a significant proportion of the range of existing plasmid size diversities in the sampled soil samples had been collected (5).

### Plasmid purification and sequencing.

S. meliloti (non-pSym) cryptic plasmids were prepared by following an isolation protocol appropriate for high-molecular-weight plasmids. Each S. meliloti LPU isolate was grown in 1 liter of tryptone-yeast (TY) medium at 28°C with shaking at 250 rpm until the late log phase of growth, and the bacterial cells were collected by centrifugation at 10,000 × *g* for 10 min for plasmid isolation. The latter step was then carried out as reported by Jouanin et al. (39). The final concentration of purified plasmid DNA was estimated by absorbance at 260 nm (NanoDrop ND 1000 Spectrophotometer). The average concentration of plasmid DNA ranged between ca. 20 and 400 ng/μl. After the purification procedure, the plasmid preparations from the different LPU isolates were mixed with comparable amounts of DNA in order to have plasmid DNA from all isolates equally represented within the final DNA pool for high-throughput nucleotide sequencing. The pooled plasmid DNA was sequenced through the use of a MiSeq (Illumina) platform at CeBiTec, Bielefeld University, Germany. The output data yielded ca. 596 Mb of DNA sequence with 50-fold coverage on average. The sequence data have been deposited with links to BioProject (see below).

### Identification of sequence reads of plasmid origin and assembly of continuous overlapping DNA sequences (contigs).

All the stretches of individual sequence reads (i.e., contigs) were assembled with Newbler v.2.6 Roche software to generate a first set. In the second step, the contigs were depurated to preserve only the information that corresponded to cryptic plasmids, discarding contigs that had originated from the sequencing of contaminating pSym and chromosomal DNA. To that end, two procedures were used. (i) First, all the contigs that displayed sequence similarity of greater than 95% to the pSyms and/or chromosomes from completely sequenced S. meliloti strains were removed. (ii) Second, the contigs with an average value for sequence coverage per base that was lower than the average value observed for the pSym and/or chromosomal sequences (+2 standard deviations [SDs]) were also removed. As expected, the higher copy number of the cryptic plasmids than of the pSyms and chromosomes, along with the purification procedure favoring the recovery of cryptic plasmids (with sizes usually between ca. 40 and 500 kb) over that of the other larger replicons, resulted in a higher level of sequence coverage per base obtained for the plasmids. While we recorded coverage of 14× and 12× in the pSym and chromosomal sequences, respectively, the coverage determined for the non-pSym-plasmid contigs was frequently more than 100× higher. On the basis of this observation, all the contigs with base coverage lower than 17× were removed. Reference values for the pSym sequence and chromosomal sequence coverages were calculated by using 40 different contigs from each of these two replicons (*cf.* Table S1 in the supplemental material). After those filtering steps were performed, the contigs longer than 1 kb were imported into the genome annotation pipeline at GenDB (40).

10.1128/mBio.00505-19.3 TABLE S1 Sequencing coverage in contigs from the Sinorhizobium meliloti chromosome and symbiotic megaplasmids (pSymA and pSymB). Download Table S1, PDF file, 0.3 MB. Copyright © 2019 López et al. 2019 López et al. This content is distributed under the terms of the Creative Commons Attribution 4.0 International license.

### Multivariate analysis of the composition of ortholog clusters in the S. meliloti replicons.

For a visual inspection of the proportions of the different categories of clusters of orthologous groups (COGs) in the various genomic compartments of S. meliloti, we performed principal-component analysis (PCA) and hierarchical clustering analysis with the amino acid sequences deduced from both the complete genomes of the S. meliloti strains listed in Table S2 (for the COGs in pSymA, pSymB, and the chromosome) and the genomes of the plasmids (deposited in GenBank; see below). The number of each COG in the different genomic compartments was estimated by means of the use of the WebMGA server (41) and the specific percent abundance calculated (Table S2). The resulting data were arranged in a matrix (replicons, COGs) that was used for the PCA and the hierarchical clustering analysis. The PCA was performed by using the Factominer package for R (42). The hierarchical clustering analysis, heat maps, and graphics were produced through the use of the pheatmap (43) and ggplot2 (44) R packages, respectively, and then edited using Inkscape software. Hierarchical clustering analysis was performed by the complete clustering method on the basis of Euclidean distances.

### Identification of chromosome and megaplasmid core genes and singletons.

The EDGAR tool (45) was used to identify core genes (i.e., those genes present in all of the strains investigated) and singletons (i.e., strain-specific genes [those unique in a given set of strains under analysis]). For these calculations, and to cover different genotypes, we selected 6 S. meliloti strains among the 21 fully sequenced rhizobia of this species. Strains 2011, AK83, BL255C, GR4, SM11, and Rm41, chosen on the basis of their different positions within the S. meliloti phylogeny, constituted the set. The core and singleton gene sets were calculated separately with EDGAR for chromosomes, pSymAs, and pSymBs. All the genomic analyses that involved S. meliloti core genes were performed through the use of the gene set of orthologs from strain 2011.

The singletons corresponded to the pooled combination of all the genes that were specific to the group of S. meliloti strains that we chose for the study (Table S3). For the identification of S. meliloti chromosomal core genes (CCs) with increasing ancestry (i.e., CC1 to CC*n* for a given conserved core), conserved-gene sets among several genomes from different species, genera, and families with increasing evolutionary distances from S. meliloti were calculated with EDGAR. In brief, CC1 to CC*n* were obtained by rescuing the corresponding core genomes of a set of bacterial chromosomes with increasing ancestry that were sequentially included throughout the evolutionary lineage presented in the phylogenetic trees illustrated in the figure attached to Table S5. The CC*n* gene sets corresponded in all instances to the set of orthologs present in S. meliloti reference strain 2011. A similar criterion was used to estimate CC10 to CC15 within the order *Rhizobiales* also (in accordance with the phylogenetic tree attached to Table S5). Each of the resulting core-gene sets corresponded to the orthologs in the bacterial species indicated in Fig. 6.

10.1128/mBio.00505-19.5 TABLE S3 List of core genes and singletons in the S. meliloti strains used in this work. Download Table S3, XLSX file, 0.9 MB. Copyright © 2019 López et al. 2019 López et al. This content is distributed under the terms of the Creative Commons Attribution 4.0 International license.

### Codon usage analysis.

Modal codon usages for different gene sets were calculated as previously described by Davis and Olsen (9). The estimation of distances between specific gene sets was performed by calculating the Manhattan distance—i.e., the sum of the absolute values of the vertical and horizontal distances among the sets—for each amino acid, followed by the calculation of multidimensional Euclidean distances as reported elsewhere (9). The uncertainty in distance values was estimated by bootstrap analyses (1,000 replicates) in which each replicate resulted from the resampling (with restitution) of each of the gene sets under consideration. Average distances and standard deviations (SDs) were thus obtained (20).

To evaluate differences between the modal codon usages of two different gene sets, i.e., sets A and B, the average distance resulting from a bootstrap calculation (with restitution performed as described above) was compared to the average distance calculated under the assumption that gene sets A and B were drawn from a common gene pool. To calculate the latter distance, gene sets A and B were pooled and then randomly divided into two new sets of sizes equal to those of the original A and B sets (1,000 replicates) (9). (Table S4 includes worksheets with the distances between specific gene sets as well as the corresponding bootstrapped and randomized data.) The SDs were estimated for all the distances calculated.

10.1128/mBio.00505-19.6 TABLE S4 Modal codon usage distances among all the Sinorhizobium meliloti gene sets characterized in this work Download Table S4, XLSX file, 0.3 MB. Copyright © 2019 López et al. 2019 López et al. This content is distributed under the terms of the Creative Commons Attribution 4.0 International license.

The statistical significance of the results of comparisons between two codon usage distances (i.e., the distance between gene sets A and B and the distance between gene sets C and D) was evaluated by using the *z*-test (for data with normal distribution) or the Mann-Whitney test (for data that were not normally distributed).

The correspondence analyses (CAs) were performed on the basis of the relative synonymous codon usages (RSCUs) as variables by the use of CodonW software (J. F. Peden, http://codonw.sourceforge.net/). To indicate the position of modal codon usages in the CA, the DNA sequences were artificially generated from modal frequencies through the use of a homemade Perl script and introduced as supplemental elements in the CA. The CA graphics were generated by the R package Ggplot2 program (44) and edited with Inkscape software. Neighbor-joining trees based on modal codon usage data were generated by software published elsewhere (9) along with the neighbor program of the PHYLIP package (46). Phylogenetic trees were visualized and edited by the use of the Figtree application (47).

### Phylogenetic analysis.

Multiple alignments of orthologous protein sets were calculated through the use of MUSCLE software (48). The aligned sequences of a given species were then concatenated into one large amino acid sequence that was employed—together with the other orthologous concatemers—to generate a phylogenetic tree by the use of the neighbor-joining method as implemented in the PHYLIP software package (46).

### S. meliloti modal tRNA adaptation index (m-tAI).

The tAI used previously for individual genes (29) was applied here to estimate the average level of efficiency with which a given gene set is recognized by the intracellular tRNA pool. The m-tAI value for a given S. meliloti core-gene set was calculated through the use of an artificially generated nucleotide sequence (*cf.* the R script in Table S7) that was designed to preserve both the modal codon usage and the composition of the translated protein product to be the same as those corresponding to the ones in the set of genes under analysis. The artificially generated polypeptide was chosen to have a length of 10,000 codons to correctly represent the modal codon usage, especially for synonymous codons from amino acids of low abundance. The calculations of the m-tAI values were performed by means of the software available at https://github.com/mariodosreis/tai together with the *s_ij_* weights provided for S. meliloti strain 1021 by Sabi and Tuller (49) and the tRNA gene copy numbers reported for the strain 2011 at http://lowelab.ucsc.edu/tRNAscan-SE/. (*s_ij_* weight values represent parameters that have been optimized to more precisely represent the efficiency of the interaction between the *i*th codon and *j*th anticodon [29]). Similar calculations were performed to obtain the m-tAI values for the core-gene sets of the bacterial species presented in Fig. 9 (*Rhizobiales*) through employment of the tRNA copy number of each of the indicated species and the *s_ij_* values determined for S. meliloti strain 1021 for all of those species.

### Quantitative composition of the S. meliloti proteome.

S. meliloti 2011 was cultured to the stationary phase of growth in the following defined medium: sucrose, 5 g per liter; MgSO_4_·7H_2_O, 0.25 g per liter; NH_4_Cl, 0.32 g per liter; MOPS, 10 g per liter; CaCl_2_·2H_2_O, 100 mg per liter; anhydrous FeCl_3_, 6 mg per liter; H_3_BO_3_, 3 mg per liter; MnSO_4_·H_2_O, 1.7 mg per liter; ZnSO_4_·7H_2_O, 0.3 mg per liter; NaMoO_4_·2H_2_O, 0.12 mg per liter; CoCl_2_·6H_2_O, 0.065 mg per liter; K_2_HPO_4_, 1 g per liter; KH_2_PO_4_, 1 g per liter; biotin, 1 mg per liter; thiamine, 10 mg per liter. A culture aliquot containing 40 optical density units was cooled on ice and centrifuged to collect the cell pellet. The proteins were extracted and separated into the cytosolic and membranous subcellular fractions according to a protocol described elsewhere (50). The proteins from both fractions were cleaned up by acetone precipitation, the pellet was digested directly with trypsin, and the resulting peptides were reduced with dithiothreitol and alkylated with iodoacetamide, as described previously (51). The tryptic peptides were separated, identified, and quantified by nanoRSLC high-performance liquid chromatography coupled to electrospray ionization (ESI)-Orbitrap mass spectrometry (Thermo Scientific, Germany), as reported earlier (52). The raw tandem mass-spectrometry (MS/MS) spectra were preprocessed and analyzed using QuPE software (53), with settings identical to those previously used (52). The peptide intensities were quantified from the mass spectrometry-derived MS^1^ precursor ion chromatograms by the use of the RelEx linear exclusion mechanism (54). The absolute abundance of each protein in the proteome was estimated by calculating the average MS^1^ signal response of the three most intensely detected unique tryptic peptides (55).

### Data accessibility.

The pooled plasmid sequence data have been deposited with links to BioProject accession number PRJEB32149 in the NCBI BioProject database (https://www.ncbi.nlm.nih.gov/bioproject/). The genomes of the plasmids determined in this work have been deposited in GenBank (see below) under the following accession numbers (for the COGs in S. meliloti plasmids): NC_004965.1, NC_010865.1, NC_013545.1, NC_019313.1, NZ_CP021796.1, NZ_CP021811.1, NZ_CP021815.1, NZ_CP021816.1, NZ_CP021817.1, NZ_CP021826.1, NZ_CP021821.1, NZ_CP021807.1, NZ_CP021832.1, NZ_CP021803.1, NC_019846.2, NC_019847.2, NC_018682.1, NZ_CP021213.1, NZ_CP021214.1, NZ_CP021215.1, and NZ_CP021216.1.
