# Supplementary figures and images for: Codon Usage Heterogeneity in the Multipartite Prokaryote Genome: Selection-Based Coding Bias Associated with Gene Location, Expression Level, and Ancestry
Source: mBio. 2019 May 28;10(3):e00505-19. doi: 10.1128/mBio.00505-19 (PMC6538778; doi:10.1128/mBio.00505-19)

FIGURE S1

A

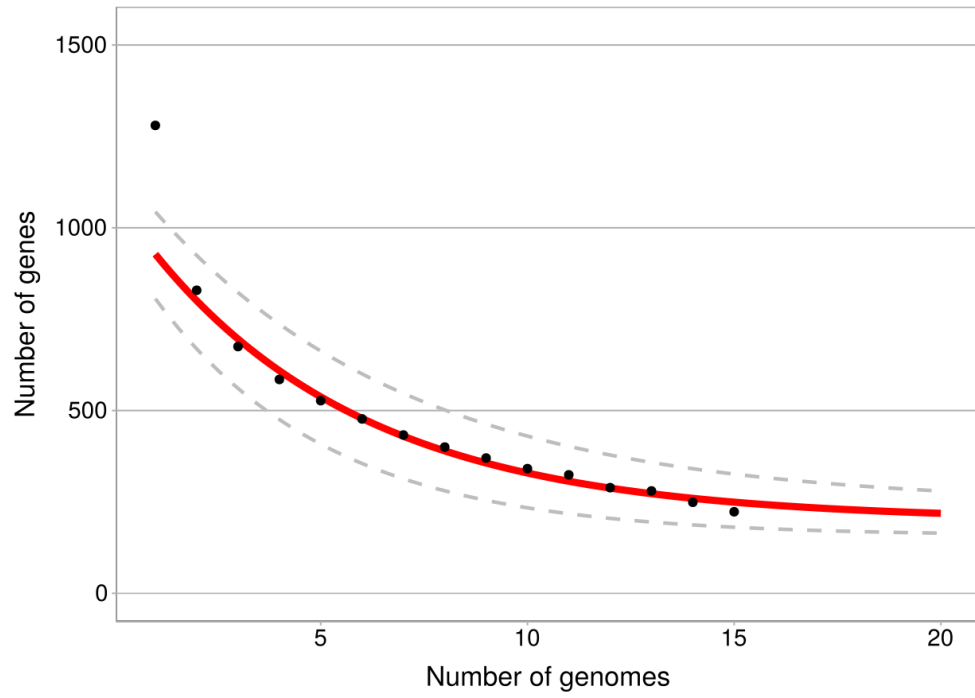

B

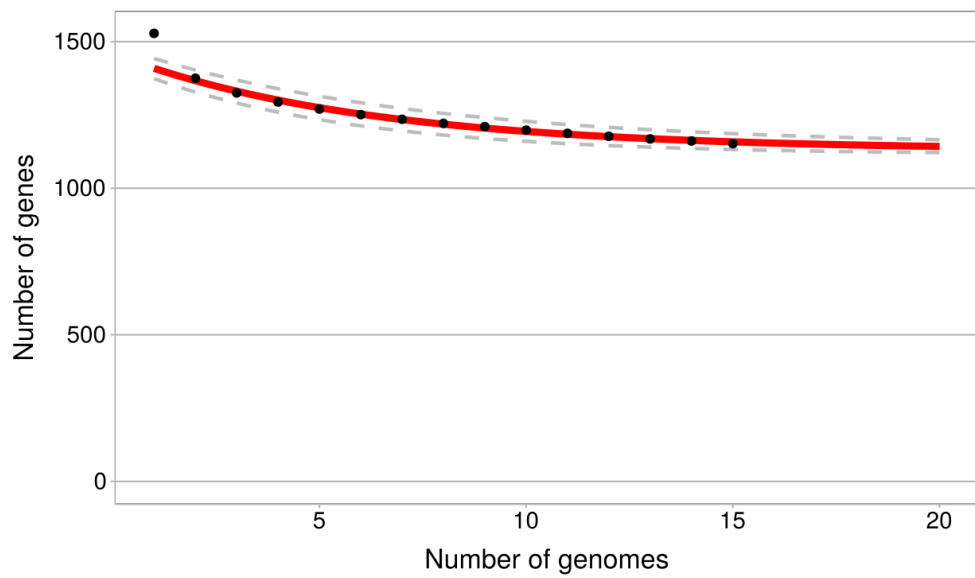

Supplement: FIG S1 [file mBio.00505-19-sf001.pdf]

FIGURE S2

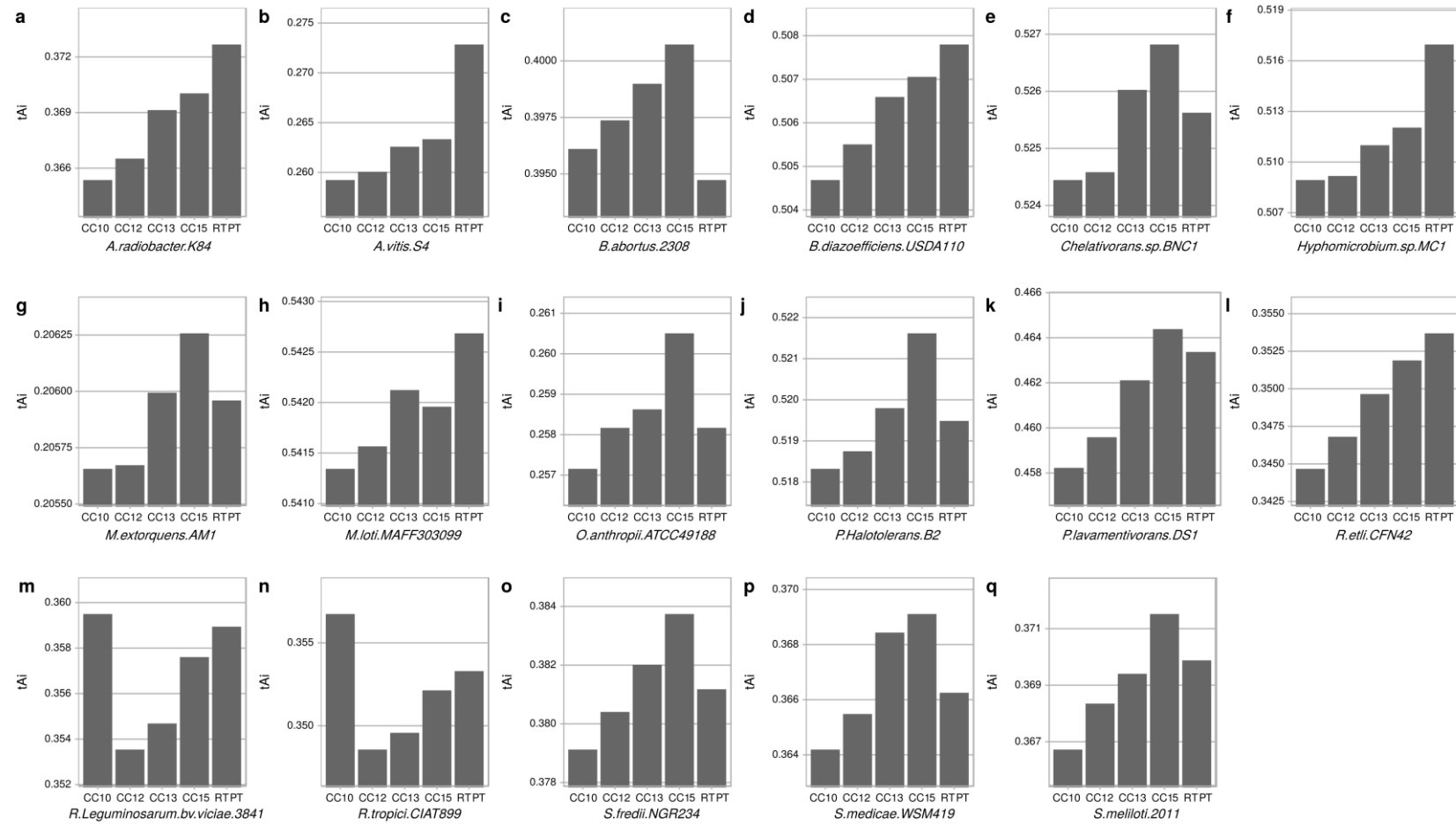

Supplement: FIG S2 [file mBio.00505-19-sf002.pdf]
