# Supplementary material for: Codon Usage Heterogeneity in the Multipartite Prokaryote Genome: Selection-Based Coding Bias Associated with Gene Location, Expression Level, and Ancestry
Source: mBio. 2019 May 28;10(3):e00505-19. doi: 10.1128/mBio.00505-19 (PMC6538778; doi:10.1128/mBio.00505-19)
Supplement: TABLE S1 [file mBio.00505-19-st001.pdf]

Table S1. Sequencing coverage in contigs from the *Sinorhizobium meliloti* chromosome and symbiotic megaplasmids (pSymA and pSymB). The contigs were selected based on the presence of known genes from the different *S. meliloti* replicons. In all instances, the sequence similarity searches through the use of BLASTn resulted in an identity of more than 99% and a coverage of more than 95%. The coverage was expressed as R/C = (number of reads / contig sequence length) x 1,000. The average coverage and standard deviation was calculated for each replicon and a cutoff value of 66 R/C (average + 2SD) used to decide whether or not a contig was of cryptic-plasmid origin. The length of each sequence read was 250 bp.

	Chromosome	pSymA	pSymB
**R/C average**	49	55	49
**standard deviation (SD)**	7,4	5,5	4,9
**average + 2SD**	64,0	65,8	59,2
**cutoff value**	**66** (larger R/C values were considered to correspond to contig from cryptic-plasmid origin)		

contig id	lenght (L, bp)	number of reads (R)	coverage (R/C)	contig origin
**contig00005**	28280	1413	50	chromosome
**contig00009**	24671	1256	51	chromosome
**contig00017**	20125	1176	58	chromosome
**contig00020**	19642	1149	58	chromosome
**contig00021**	19397	935	48	chromosome
**contig00022**	19220	1110	58	chromosome
**contig00023**	19073	1030	54	chromosome
**contig00027**	18527	991	53	chromosome
**contig00028**	18473	832	45	chromosome
**contig00032**	18081	1004	56	chromosome
**contig00034**	17911	979	55	chromosome
**contig00035**	17807	744	42	chromosome
**contig00039**	17054	975	57	chromosome
**contig00049**	15975	747	47	chromosome
**contig00060**	15398	774	50	chromosome
**contig00066**	14406	742	52	chromosome
**contig00074**	13662	648	47	chromosome
**contig00081**	13282	587	44	chromosome
**contig00094**	12413	612	49	chromosome
**contig00208**	8476	374	44	chromosome
**contig00212**	8406	372	44	chromosome
**contig00213**	8387	541	65	chromosome
**contig00215**	8337	353	42	chromosome
**contig00218**	8276	414	50	chromosome
**contig00223**	8179	399	49	chromosome
**contig00222**	8174	532	65	chromosome
**contig00224**	8147	315	39	chromosome
**contig00228**	8077	400	50	chromosome
**contig00229**	8039	342	43	chromosome
**contig00232**	8021	424	53	chromosome
**contig00234**	7976	327	41	chromosome

**contig00235**	7949	527	66	chromosome
**contig00236**	7946	381	48	chromosome
**contig00237**	7939	472	59	chromosome
**contig00238**	7930	313	39	chromosome
**contig00239**	7925	396	50	chromosome
**contig00240**	7918	305	39	chromosome
**contig00242**	7901	378	48	chromosome
**contig00243**	7886	328	42	chromosome
**contig00246**	7804	346	44	chromosome
**contig00248**	7772	301	39	chromosome
**contig00249**	7768	316	41	chromosome
**contig00250**	7716	302	39	chromosome
**contig00252**	7665	319	42	chromosome
**contig00259**	7601	394	52	chromosome
**contig00258**	7595	433	57	chromosome
**contig00018**	20046	999	50	pSymA
**contig00019**	19961	1058	53	pSymA
**contig00026**	18579	1010	54	pSymA
**contig00040**	17035	981	58	pSymA
**contig00054**	15825	832	53	pSymA
**contig00063**	15100	815	54	pSymA
**contig00068**	14108	779	55	pSymA
**contig00071**	13943	737	53	pSymA
**contig00073**	13717	597	44	pSymA
**contig00078**	13511	744	55	pSymA
**contig00083**	13199	718	54	pSymA
**contig00087**	13028	694	53	pSymA
**contig00105**	11414	671	59	pSymA
**contig00122**	10917	455	42	pSymA
**contig00130**	10650	568	53	pSymA
**contig00144**	10136	628	62	pSymA
**contig00147**	10012	624	62	pSymA
**contig00153**	9827	540	55	pSymA
**contig00155**	9810	460	47	pSymA
**contig00158**	9742	564	58	pSymA
**contig00164**	9544	535	56	pSymA
**contig00168**	9405	670	71	pSymA
**contig00194**	8829	543	62	pSymA
**contig00199**	8699	498	57	pSymA
**contig00200**	8664	552	64	pSymA
**contig00202**	8623	406	47	pSymA
**contig00201**	8613	446	52	pSymA
**contig00205**	8583	468	55	pSymA
**contig00216**	8278	439	53	pSymA
**contig00217**	8253	468	57	pSymA
**contig00230**	8030	465	58	pSymA
**contig00231**	8028	462	58	pSymA
**contig00244**	7856	409	52	pSymA
**contig00245**	7834	391	50	pSymA
**contig00256**	7655	407	53	pSymA
**contig00265**	7500	390	52	pSymA
**contig00271**	7434	433	58	pSymA
**contig00007**	26147	1387	53	pSymB
**contig00010**	24008	1369	57	pSymB

**contig00015**	20994	898	43	pSymB
**contig00029**	18221	1000	55	pSymB
**contig00033**	18008	833	46	pSymB
**contig00036**	17617	927	53	pSymB
**contig00042**	16925	768	45	pSymB
**contig00046**	16431	832	51	pSymB
**contig00051**	15874	815	51	pSymB
**contig00055**	15670	729	47	pSymB
**contig00069**	14069	757	54	pSymB
**contig00076**	13544	577	43	pSymB
**contig00085**	13139	661	50	pSymB
**contig00091**	12484	554	44	pSymB
**contig00097**	12016	596	50	pSymB
**contig00104**	11446	506	44	pSymB
**contig00106**	11307	557	49	pSymB
**contig00111**	11195	562	50	pSymB
**contig00113**	11170	511	46	pSymB
**contig00117**	11067	518	47	pSymB
**contig00129**	10746	491	46	pSymB
**contig00131**	10623	517	49	pSymB
**contig00136**	10405	481	46	pSymB
**contig00140**	10206	516	51	pSymB
**contig00150**	9899	467	47	pSymB
**contig00163**	9544	434	45	pSymB
**contig00166**	9510	461	48	pSymB
**contig00173**	9307	437	47	pSymB
**contig00190**	8910	545	61	pSymB
**contig00192**	8877	490	55	pSymB
**contig00193**	8864	398	45	pSymB
**contig00196**	8812	401	46	pSymB
**contig00197**	8756	510	58	pSymB
**contig00203**	8611	398	46	pSymB
**contig00204**	8583	522	61	pSymB
**contig00206**	8562	409	48	pSymB
**contig00209**	8461	419	50	pSymB
**contig00210**	8435	339	40	pSymB
**contig00211**	8412	469	56	pSymB
**contig00214**	8375	382	46	pSymB
**contig00233**	7995	437	55	pSymB
**contig00251**	7681	409	53	pSymB
**contig00253**	7669	377	49	pSymB
